# Macrophage Migration Inhibitory Factor protects cancer cells from immunogenic cell death and impairs anti-tumor immune responses

**DOI:** 10.1371/journal.pone.0197702

**Published:** 2018-06-04

**Authors:** Kristen N. Balogh, Dennis J. Templeton, Janet V. Cross

**Affiliations:** Department of Pathology, University of Virginia School of Medicine, Charlottesville, VA, United States of America; University of Tennessee Health Science Center, UNITED STATES

## Abstract

The Macrophage Migration Inhibitory Factor (MIF) is an inflammatory cytokine that is overexpressed in a number of cancer types, with increased MIF expression often correlating with tumor aggressiveness and poor patient outcomes. In this study, we aimed to better understand the link between primary tumor expression of MIF and increased tumor growth. Using the MMTV-PyMT murine model of breast cancer, we observed that elevated MIF expression promoted tumor appearance and growth. Supporting this, we confirmed our previous observation that higher MIF expression supported tumor growth in the 4T1 murine model of breast cancer. We subsequently discovered that loss of MIF expression in 4T1 cells led to decreased cell numbers and increased apoptosis *in vitro* under reduced serum culture conditions. We hypothesized that this increase in cell death would promote detection by the host immune system *in vivo*, which could explain the observed impairment in tumor growth. Supporting this, we demonstrated that loss of MIF expression in the primary tumor led to an increased abundance of intra-tumoral IFNgamma-producing CD4+ and CD8+ T cells, and that depletion of T cells from mice bearing MIF-deficient tumors restored growth to the level of MIF-expressing tumors. Furthermore, we found that MIF depletion from the tumor cells resulted in greater numbers of activated intra-tumoral dendritic cells (DCs). Lastly, we demonstrated that loss of MIF expression led to a robust induction of a specialized form of cell death, immunogenic cell death (ICD), *in vitro*. Together, our data suggests a model in which MIF expression in the primary tumor dampens the anti-tumor immune response, promoting tumor growth.

## Introduction

The Macrophage Migration Inhibitory Factor (MIF) was first described in the 1960’s as a T cell secreted factor capable of inhibiting the random migration of macrophages *in vitro* [1,2]. MIF has since been characterized as an inflammatory cytokine implicated in a number of diseases, including colitis and arthritis [3,4]. Moreover, MIF has been broadly implicated in cancer, with overexpression shown in a number of solid tumor types [5–9]. Importantly, MIF overexpression in the serum of cancer patients and in tumor biopsies has been correlated with enhanced tumor progression and metastasis [10–13].

The MIF protein has an enzymatic activity, functioning as a keto-enol tautomerase, with the N-terminal proline required for this activity [14]. While no physiological substrate has been discovered, our work and the work of others have indicated the biological importance of the enzymatic activity through use of point mutations and/or inhibitors that specifically target the active site of MIF [15–17]. However, this conclusion remains controversial, as others have suggested that the enzymatic activity is dispensable for at least some of the biological functions of MIF [18,19].

Recently, several studies, including our own, strongly suggest that MIF exerts its pro-tumorigenic effects through modulation of the immunosuppressive tumor microenvironment. We published previously that, in the 4T1 model of breast cancer, MIF expression promotes tumor growth only in a host with a fully intact immune system capable of mounting an adaptive immune response [17]. Work by several other groups has shown that MIF expression suppresses dendritic cell (DC) maturation *in vitro*, as well as dampens T cell activation *in vivo* [20–22]. This suggests that MIF is an important mediator in establishment of an immunosuppressive tumor microenvironment.

One mechanism to overcome immunosuppression in the context of the tumor microenvironment is the induction of a specialized type of cell death in cancer cells termed immunogenic cell death (ICD). As recently reviewed by Dudek *et al*, this type of cell death can be induced specifically in cancer cells through treatment with certain classes of chemotherapeutics, radiation therapy, and photodynamic therapy (PDT) [23]. Cancer cells undergoing ICD exhibit cellular release of ATP and exposure of certain ER chaperones on the cell surface, including calreticulin (CALR) and HSP70 [24–27]. Expression of these molecules on even a low number of cancer cells can be recognized by the host immune system through several mechanisms, leading to a robust anti-tumor immune response as well as immunological memory against the tumor [28].

In this study, we demonstrate that depletion of MIF expression in the 4T1 model of breast cancer strongly promotes ICD *in vitro* under serum-free conditions. We present evidence supporting a model in which depletion of MIF expression in the primary tumor *in vivo* leads to a robust anti-tumor immune response marked by enhanced DC maturation, followed by increased IFNgamma-producing T cells in the tumor. This leads to greater tumor control in MIF-depleted tumors, a phenotype that we show is recapitulated in a spontaneous, genetic model of breast cancer.

## Materials and methods

### Cell lines

The 4T1 cell line was obtained directly from Caliper Life Sciences. The 4T1 cell line tested negative for mycoplasma at the University of Virginia, most recently on May 11, 2017. Cells were cultured under the conditions recommended by the ATCC. All cell lines were cultured no more than 10 passages before use in *in vivo* or *in vitro* experiments. The method for the generation of the MIF knock-down (MIF KD) 4T1 cells was published previously [17]. For reconstituted cell lines, the coding region for wild-type (WT) human MIF or mutant human P2G MIF was inserted into the pQCXI-neo vector and was used to generate retroviruses. MIF-depleted 4T1 cells were infected with WT MIF or P2G MIF-expressing viruses (or empty vector) and selected with 500ug/mL neomycin. Efficient MIF depletion and subsequent re-expression of MIF in the reconstituted cell lines was confirmed by immunoblot (Santa Cruz, #sc-20121) **(S1A and S1B Fig)**.

### Antibodies and flow cytometry

For immunoblotting, anti-cleaved caspase 3 (Cell Signaling, #9661S) was used, as well as anti-tubulin (Sigma, #T9026) as a housekeeping control. For flow cytometry analysis, cells were stained with propidium iodide (PI) (eBiosciences), Live/Dead Fixable Yellow Dead Cell Stain (Invitrogen), as well as with the list of antibodies shown in **Table 1** according to the manufacturers recommendations. All gating on cell surface markers was based on fluorescence minus on (FMO) controls. The cells were analyzed with the Beckman Coulter CyAN ADP LX 9 Color Flow Cytometer.

**Table 1:** 

Antibody Name	Fluor	Vendor	Clone
Annexin V	FITC	BioLegend	N/A
Cleaved Caspase 3	PE	Cell Signaling	5A1E
CD3e	FITC	eBioscience	145-2C11
CD8a	efluor-450	eBioscience	53–6.7
CD4	APC efluor780	eBioscience	GK1.5
IFNgamma	APC	Biolegend	XMG1.2
CD45	PerCP	BD Bioscience	30-F11
CD11b	Pacific Blue	Invitrogen	M1/70.15
CD11c	APCCy7	BioLegend	N418
CD8a	Pacific Orange	Invitrogen	5H10
CD103	FITC	BioLegend	2E7
MHCII	PE	eBioscience	M5/114.15.2
CD86	PECy7	BioLegend	GL-1
CD40	APC	BioLegend	3/23
Calreticulin	Alexafluor647	AbCam	EPR3924
HSP70	PE	Miltenyi Biotec	REA349
CD16/32 (blocking)	N/A	BioLegend	93

### Mouse tumor models and tumor dissociation

Wild-type (WT) male C57Bl/6 MMTV-PyMT mice were crossed with established female MIF knock-out (MIF KO) C57Bl/6 mice generated as described previously [29] in order to generate both WT and MIF KO MMTV-PyMT lines. Both parental strains used in the cross were of 100% C57Bl/6 background. MIF status was confirmed by PCR, with a heterozygous mouse shown as a control **(S2A Fig, top)**. Presence of the MMTV-PyMT transgene was also confirmed using PCR, with a mouse negative for the transgene shown as a control **(S2A Fig, bottom)**. Primers used for genotyping were as follows: murine MIF (F:5’ACGCAGCGCGCTCTCATAGACCAG G3’ R:3’GGTCTCTTATAAACCATTTATTTCTCC5’), Neo (F:5’TGCTCCTGCCGAGAAAG TATCCATCATGGC3’ R:3’CGCCAAGCTCTTCAGCAATATCACGGGTAG5’) and murine MMTV (F:5’TGTGCACAGCGTGTATAATCC3’ R:3’CAGAATAGGTCGGGTTGCTC5’). Immunoblot of lung tissue from WT and MIF KO mice was performed to confirm loss of MIF expression after crossing with the MMTV-PyMT line **(S2B Fig)**. Homozygous WT and MIF KO mice were monitored for the presence of mammary tumors starting at 6 weeks of age.

Female 16–18 gram BALB/c mice were purchased from Charles River Laboratories. 1.0 x 10^4^ WT or MIF KD 4T1 cells were injected into the mammary fat pad and monitored every other day for tumor growth starting 7 days after tumor implant. One hundred percent of mice develop tumors in this model using this approach. All animal studies were conducted in accordance with the University of Virginia Animal Care and Use Committee (ACUC) under protocol approval #4039 and all efforts were made to minimize suffering of animals in all experiments.

Using calipers, tumor volumes were estimated from two perpendicular measurements using the formula V = 0.4 x L x W^2^. Tumors were excised from the mammary fat pad, weighed, and then digested with 10,000 U collagenase I (Worthington Biochemical) for 60 min at 37°C, followed by addition of 30U of DNAse (Qiagen) for 10 minutes at RT. Cell suspensions were strained through a 70-um screen before use in experiments.

### Ex vivo T cell stimulation

On day 10 of 4T1 tumor growth, tumors were excised and digested as described above. The digested material was incubated in triplicate *in vitro* for 4 hours with brefeldin A (BFA) (eBioscience) in anti-CD3-coated (eBioscience) 96-well plates. Cells were then stained by flow cytometry for extracellular T cell surface markers followed by intracellular staining for IFNgamma.

### T cell depletion

Beginning two days before 4T1 cell tumor implantation, mice were treated with intraperitoneal injection of an initial dose of 200ug/mouse of anti-CD4 (clone GK1.5, BioXCell) and anti-CD8 (clone 2.43, BioXCell) antibodies in PBS, followed by similar dosing with 100ug/mouse every 4 days throughout the course of tumor growth. Tumors were excised on day 20 and weighed.

### Intratumoral and lymph node dendritic cell (DC) analysis

On day 8 of 4T1 tumor growth, tumors were excised and digested as described above. Draining inguinal and non-draining axillary lymph nodes were removed, manually dissociated using a blade, and strained through a 70-um screen. The digested/dissociated material was stained by flow cytometry for expression of dendritic cell surface markers.

### Growth curve

5.0 x 10^4^ 4T1 WT or MIF KD 4T1 cells were plated in triplicate in 6-well dishes in RPMI media containing 10% fetal bovine serum (FBS) (HyClone). After 24 hours, medium was washed off and replaced with 1% serum or serum-free RPMI. The number of cells per well was counted each day up to three days after culture in 1% or serum-free media by hemocytometer.

### Cell death and ICD cell surface marker analysis

5.0 x 10^4^ WT or MIF KD 4T1 cells were plated in triplicate in 6-well dishes in 10% serum-containing RPMI. After 24 hours, media was washed off and replaced with serum-free RPMI. After a further 48 hours in serum-free media, cells were harvested and stained for Annexin V and propidium iodide (PI), cleaved caspase 3 or calreticulin and HSP70 by flow cytometry, or lysed in Laemmli sample buffer for cleaved caspase 3 analysis by immunoblot.

### ATP assay

5.0 x 10^4^ WT or MIF KD 4T1 cells were plated in triplicate in 6-well dishes in 10% serum-containing RPMI. After 24 hours, media was washed off and replaced with serum-free RPMI. After a further 24 hours, media was sampled by removing 100uL from each well. ATP was measured in the media using the ATP Bioluminescence Assay Kit HS II (Roche) and the concentration was determined by comparing to a standard curve.

### Statistical analysis

Data are presented as mean +/- SEM. Data was analyzed either by Student’s t-test or one-way ANOVA using the Graph-Pad Prism analysis software. P values are represented in the figures as * p<0.05, ** p<0.01, *** p<0.001, **** p<0.0001.

## Results

### MIF expression promotes tumor progression in the MMTV-PyMT and 4T1 models of breast cancer

**Fig 1:**
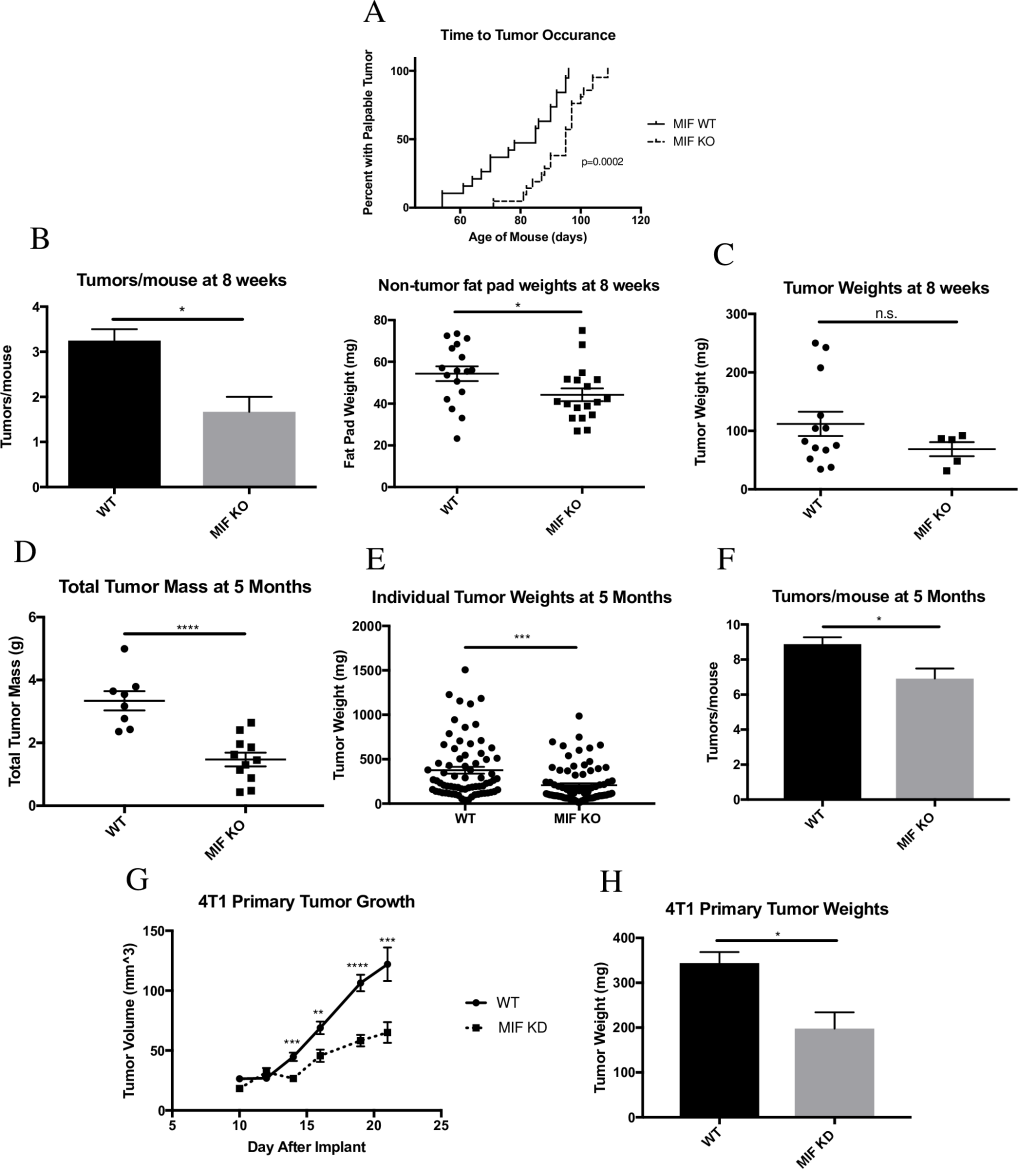
MIF expression promotes tumor progression in the MMTV-PyMT and 4T1 models of breast cancer. Female WT and MIF KO MMTV-PyMT mice were monitored starting at 6 weeks of age for the presence of mammary tumors. **A,** Age was recorded on the day of appearance of the first tumor. n = 19 for WT mice, n = 21 for MIF KO mice. **B-C,** Mice were euthanized at 8 weeks of age. Detectable tumor material and non-tumor bearing fat pads were removed, weighed, and enumerated. Each data point on dot plots represents one individual fat pad or tumor, with some mice having multiple tumors. n = 4 mice for WT and n = 3 mice for MIF KO. **D-F,** Mice were euthanized at 5 months of age. Tumors were removed from the fat pads, enumerated, and weighed. Each data point in **(D)** represents one mouse. Each data point in **(E)** represents one tumor. n = 8 mice for WT, n = 11 mice for MIF KO. **G,** 1.0 x 10^4^ WT or MIF KD 4T1 cells were implanted in the mammary fat pad of female Balb/c mice and tumor size was monitored starting at day 10 by caliper measurement. **H,** Tumors were harvested at day 22 of tumor growth post implantation and weighed. Data in **G** and **H** are representative of three independent experiments, with n = 5 mice/group in each experiment. One-way ANOVA. *p<0.05, ** p<0.01, *** p<0.001 **** p<0.0001.

To study the role of MIF in a syngeneic model of breast cancer, we utilized the MMTV-PyMT murine model of spontaneous breast cancer. In this model, the polyoma virus middle T oncogene is expressed under the transcriptional control of the mouse mammary tumor virus (MMTV) promoter, leading to development of adenocarcinomas in multiple mammary fat pads of female mice carrying the transgene [30]. This model closely mimics the progression of human breast cancer, advancing from hyperplasia through adenocarcinoma over a time course of approximately five months after birth [31]. The primary tumors also spontaneously metastasize to the lungs, as is often seen in human breast cancer progression [30]. To examine the role of MIF in tumor growth using this model, we compared female MIF-expressing (WT) and MIF-deficient (MIF KO) MMTV-PyMT transgenic mice. The average time to tumor occurrence was significantly delayed in MIF KO mice **(Fig 1A)**. When comparing age-matched 8-week-old mice, the point at which about half of the mice in our studies presented with at least one palpable tumor mass, MIF KO mice showed significantly fewer tumors per mouse and, in fat pads that did not yet harbor detectable tumors, smaller fat pads by weight **(Fig 1B)**. While these fat pads did not have palpable tumor material at this time point, these fat pads likely harbor early lesions and/or sub-detectable tumors, based on previous work in this model [31]. The decreased fat pad weight in the MIF KO mice suggests that they contain a reduced burden of these earlier tumorigenic lesions when compared to the MIF expressing mice. While the MIF KO mice had fewer tumors per mouse, when comparing individual tumor sizes there was no statistically significant difference between WT and MIF KO mice at this early time point **(Fig 1C)**. When comparing late-stage, age-matched 5-month-old mice, MIF KO mice had significantly less total tumor burden compared to WT mice **(Fig 1D)**, as well as fewer tumors at the largest end of the size distribution **(Fig 1E)**. MIF KO mice also developed significantly fewer tumors per mouse when compared to WT mice by 5 months of age **(Fig 1F)**. Together, these data suggest that loss of MIF expression in the MMTV-PyMT model leads to a delay in tumor appearance and reduction in tumor growth.

While the MMTV-PyMT model is a valuable tool due to its similarity to human disease progression, technical limitations of the model complicate mechanistic studies due to the long tumor growth period and difficulties in synchronizing tumor development. In addition, since MIF expression is absent in the whole animal rather than just in the tumor, it is not possible to determine the role of tumor cell-derived MIF using this model. We therefore moved to the more tractable 4T1 model of breast cancer to dissect the contributions of MIF to tumor growth. The 4T1 cell line is an orthotopic model of triple-negative breast cancer syngeneic to Balb/c mice [32], in which tumors spontaneously metastasize to the lungs, bone, brain and liver, similar to the metastatic profile seen in human breast cancer [33]. We have previously demonstrated that depletion of MIF in the 4T1 model results in delayed tumor growth and impaired metastasis [17]. To confirm the tumor growth observations, MIF knock-down (MIF KD) and MIF-expressing (WT) 4T1 cells were implanted in the mammary fat pad of Balb/c mice, and tumor growth was monitored over the course of 21 days. As in the MMTV-PyMT model, and consistent with our previous work, loss of MIF expression in the primary tumor reduced tumor growth in the 4T1 model **(Fig 1G and 1H).**

### MIF expression promotes cell growth and protects against cell death in vitro in serum-free conditions

**Fig 2:**
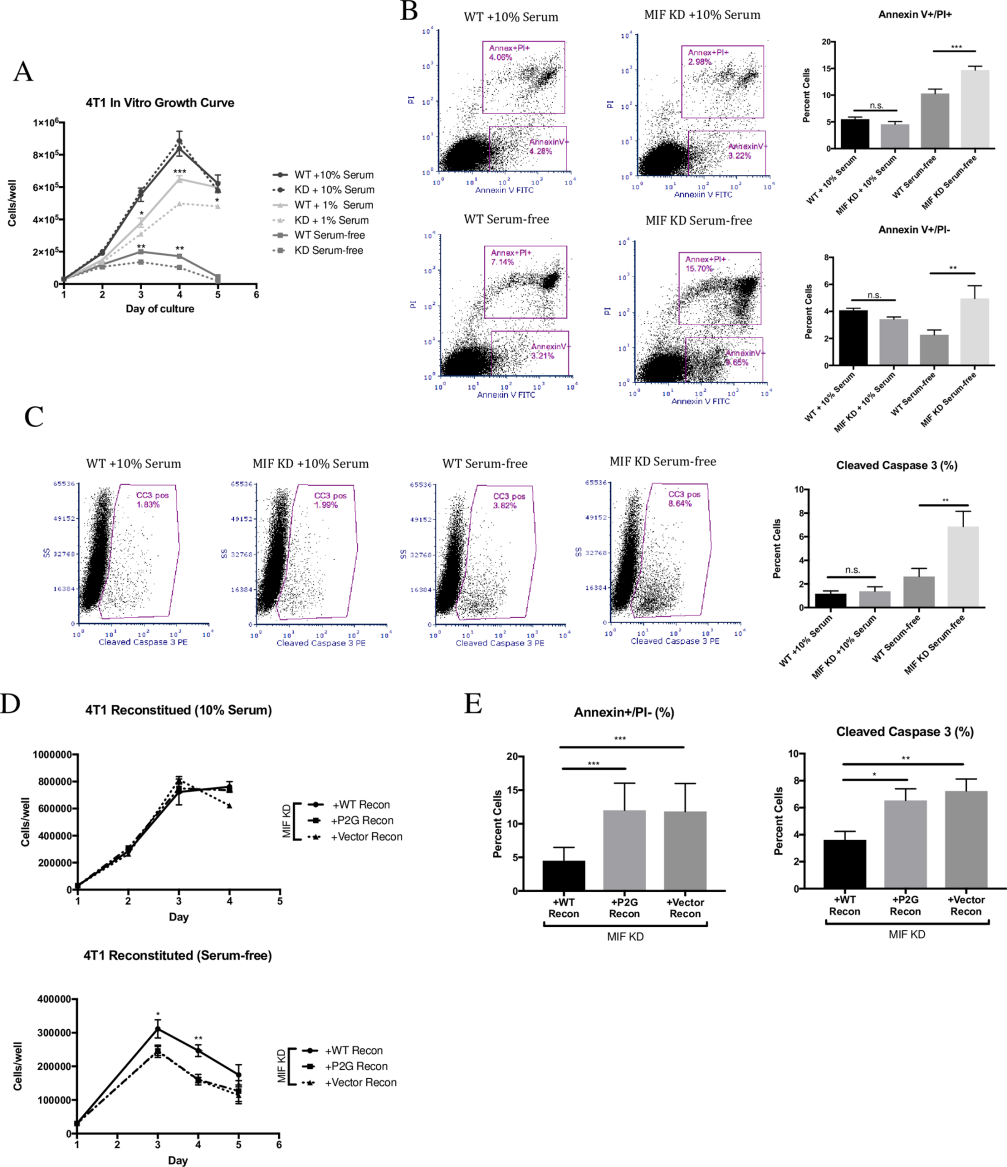
MIF expression promotes cell growth and protects against cell death in vitro in serum-free conditions. **A,** WT or MIF KD 4T1 cells were grown in 10% serum-containing media overnight, and then switched to fresh 10%, serum, 1% serum, or serum-free media. Cells were counted using a hemocytometer every day for 3 days. Determination of the slope of the lines using best-fit analysis of the curves during the exponential growth phase for each condition demonstrates that growth rate of the WT cells is greater than that of the MIF KD in both 1% serum (WT = 250417 +/- 11333 vs MIF KD = 181111 +/- 3836) and serum-free conditions (WT = 85000 +/- 3368 vs MIF KD = 53056 +/- 13391). Moreover, the mean cell number at the curve peak is higher in the WT cultures in both 1% (WT = 650000+/-20367 vs MIF KD = 497778+/-12814 at day 4) and serum free conditions WT = 200000+/-5774 vs MIF KD = 136111+/-14087 at day 3). **B,** WT or MIF KD 4T1 cells were grown in 10% serum-containing media overnight, and then switched to fresh 10% or serum-free media for a further 48 hours. Cells were then stained for Annexin V and PI using flow cytometry, or **C,** stained for cleaved caspase 3 by flow cytometry. **D,** MIF KD 4T1 cells were reconstituted (recon) with WT MIF, P2G MIF or with an empty vector as a control and were grown in 10% serum-containing media overnight, and then switched to fresh 10% media (top) or serum-free media (bottom) and counted using a hemocytometer every day for 3 days. Examination of the growth rate as in Panel A demonstrates that reconstitution with WT MIF (but not the P2G mutant) rescued both the growth rate (+WT Recon = 140676 vs +P2G Recon = 108000, +Vector Recon = 107491) and the peak cell number (+WT Recon = 311352+/-27168 vs +P2G Recon = 246000+/-13443, +Vector Recon = 244981+/-18604) in serum free growth conditions. **E,** After 48 hours in fresh 10% or serum-free media, cells were stained for Annexin V and PI (left) or cleaved caspase 3 (right) using flow cytometry. Data is the mean of 3 independent experiments with 3 replicates per experiment. One-way ANOVA. * p<0.05, ** p<0.01, *** p<0.001.

We previously reported that the *in vitro* growth of 4T1 cells was not altered following depletion of MIF under standard cell culture conditions with 10% serum [17]. In order to more closely mimic the more nutrient-deficient microenvironment murine tumor cells might experience upon orthotopic implant into the mammary fat pad, WT or MIF KD 4T1 cells were cultured *in vitro* in 1% serum or serum-free media. Cells were counted each day for 3 days after the media was changed, and compared to standard 10% serum growth conditions. As expected, equal numbers of WT and MIF KD cells were observed when cultured in 10% serum **(Fig 2A)**. However, when the serum concentration was reduced to 1% or the cells were cultured in serum-free media, the MIF KD cells showed a significant reduction in cell number when compared to the WT cells **(Fig 2A)**.

To determine if the decreased cell numbers in the MIF KD cultures under serum-free conditions was due to increased cell death, we measured this using several approaches. Annexin V and propidium iodide (PI) co-staining revealed that MIF-depletion led to an increased abundance of cells undergoing both early apoptosis (Annexin V+/PI-) and late apoptosis/necrosis (Annexin V+/PI+) after 48 hours in serum-free media. In contrast, no differences were observed when the WT and MIF KD cells were cultured in 10% serum **(Fig 2B)**. Cleaved caspase-3, a marker of late apoptosis, was also increased in the MIF KD cells after 48 hours in serum-free conditions, as detected by both flow cytometry **(Fig 2C)** and immunoblot **(S3 Fig)**.

To confirm that the reduction in cell numbers observed in the MIF KD cultures was indeed due to loss of MIF expression, we reconstituted the MIF KD cells with WT MIF. In parallel, to determine whether the enzymatic activity of MIF is involved in this phenotype, we reconstituted the MIF KD cells with the enzymatically inactive point mutant form of MIF (MIF P2G). MIF KD cells engineered to re-express WT MIF, but not P2G MIF, led to increased cell numbers in serum-free conditions **(Fig 2D, bottom)**. As expected, no difference in cell number was seen between these three cell lines in 10% serum **(Fig 2D top)**. These data confirm that loss of MIF expression leads to a disadvantage when cells are cultured in low serum conditions, and demonstrates the requirement for the enzymatic tautomerase activity for this biological function. Similarly, reconstitution of MIF KD cells with WT MIF only (but not P2G MIF) reduced the cell death observed in serum-free conditions **(Fig 2E)**. Taken together, these results show that MIF-depletion leads to increased cell death when cells are cultured under serum-free conditions.

### MIF expression in the primary tumor dampens anti-tumor T cell responses in vivo

**Fig 3:**
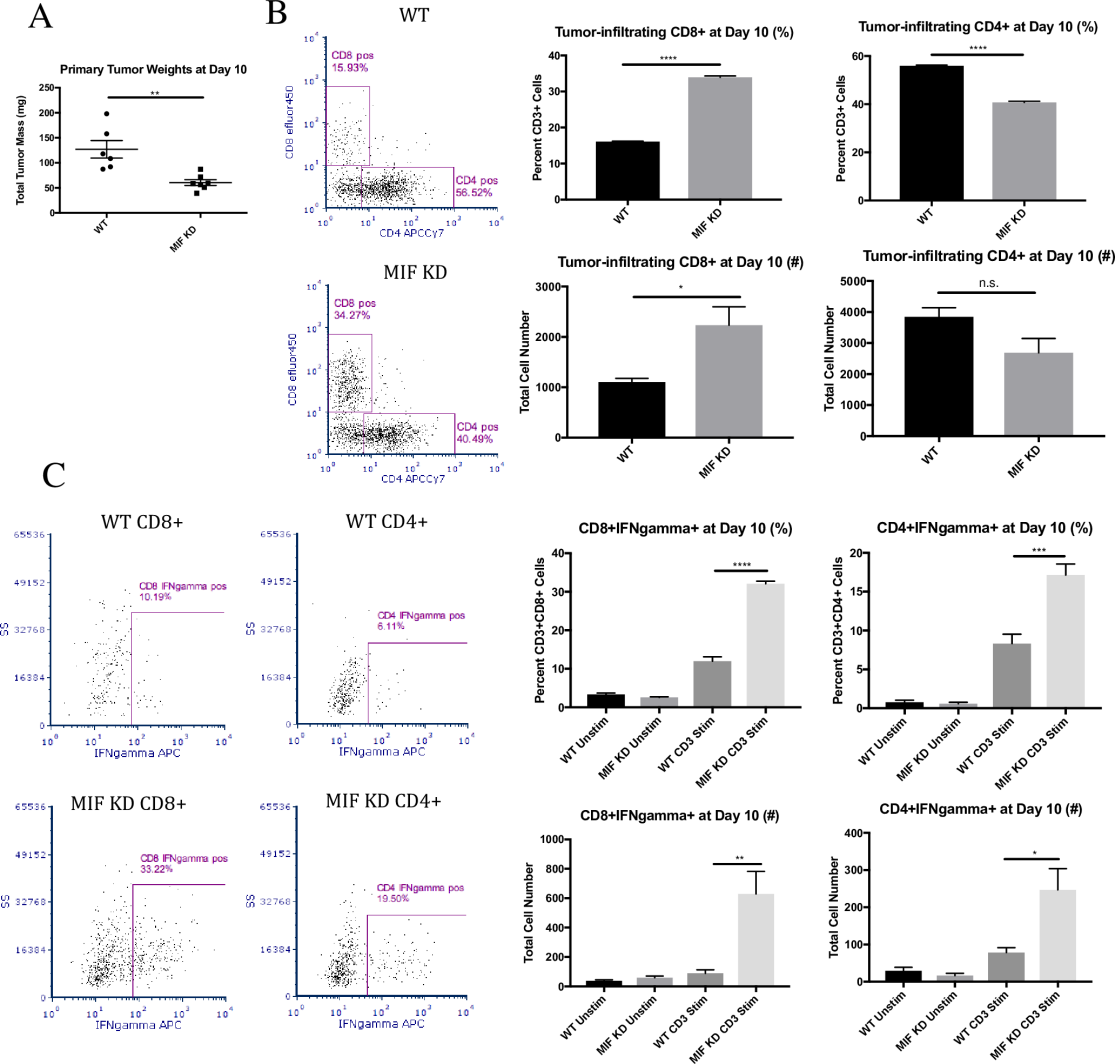
MIF expression in the primary tumor dampens anti-tumor T cell responses in vivo. 1.0 x 10^4^ WT or MIF KD 4T1 cells were implanted in the mammary fat pad of female Balb/c mice. **A,** Tumors were harvested at day 10 of tumor growth and weighed. Tumors were then digested and dissociated, and cells were cultured *in vitro* for 4 hours in the presence of BFA +/- anti-CD3 stimulation. Cells were stained for CD4 and CD8 surface expression **(B)** and intracellular IFNgamma **(C)** by flow cytometry. Data shown are representative of one of three independent experiments, with n = 6 mice/group. One-way ANOVA. * p<0.05, ** p<0.01, ***p<0.001, **** p<0.0001.

A recent report suggests that cancer cells that are dying due to cellular stress can elicit a strong anti-tumor immune response [34]. Therefore, we next asked whether loss of MIF expression was associated with a heightened anti-tumor immune response *in vivo*. We analyzed T cell infiltration and activation in day 10 WT and MIF KD 4T1 tumors. This time point was selected because it is the earliest time point at which a statistically significant difference in tumor size between WT and MIF KD tumors is detectable **(Fig 3A)**. MIF KD tumors contained significantly more tumor-infiltrating CD8+ T cells, both as a percent of total CD3+ T cells and by absolute cell number **(Fig 3B)**. The MIF KD tumors also contained significantly fewer CD4+ T cells by percent. However, no significant difference was observed in the total number of CD4+ T cells **(Fig 3B)**. Interestingly, both populations of tumor-infiltrating T cells in the MIF KD tumors were more activated, as exhibited by the ability of both CD8+ and CD4+ T cells to express IFNgamma after *ex vivo* re-stimulation **(Fig 3C)**. The gating strategy for the described T cell analysis is shown in S4 Fig, and the placement of all gates was determined based on the fluorescence minus one (FMO) controls shown **(S4 Fig)**. Collectively, these results suggest that loss of MIF expression in the primary tumor results in an enhanced early anti-tumor T cell response, leading to decreased tumor outgrowth.

### Systemic depletion of CD4+ and CD8+ T cells restores MIF KD tumor growth in vivo

**Fig 4:**
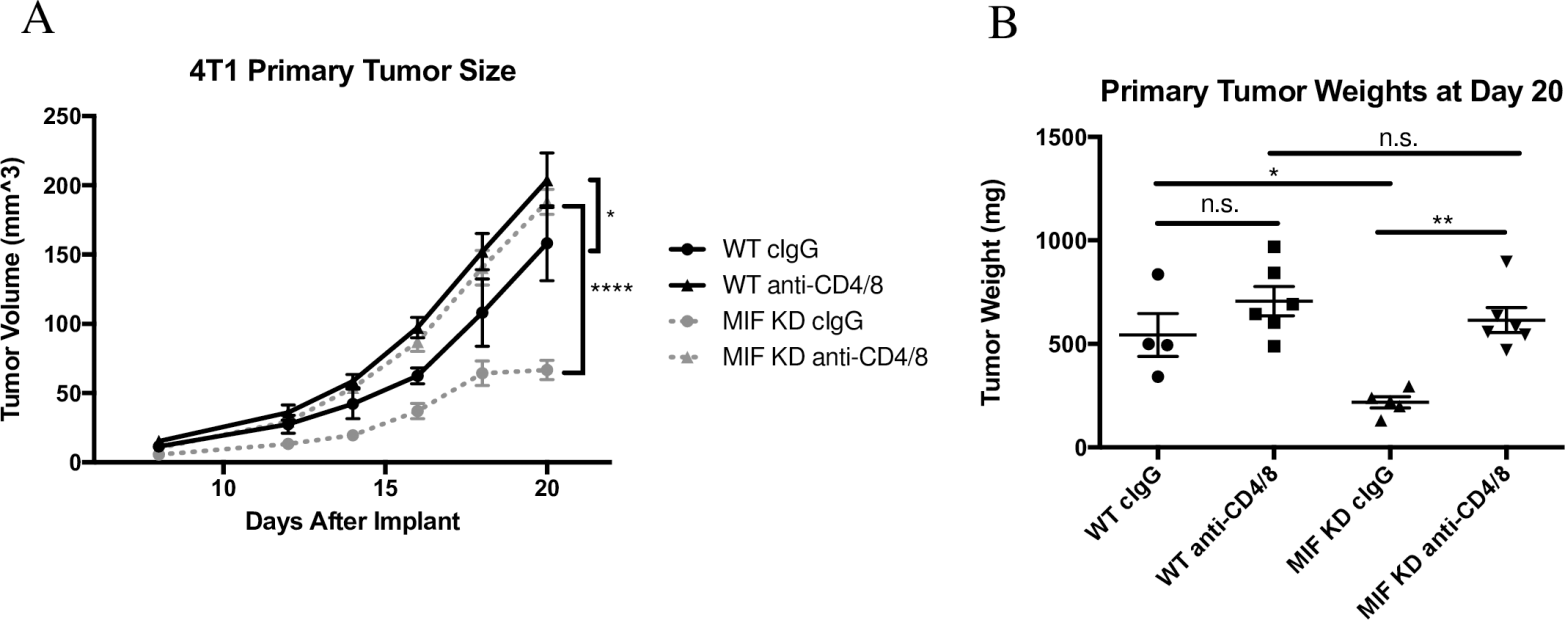
Systemic depletion of CD4+ and CD8+ T cells restores MIF KD tumor growth in vivo. 1.0 x 10^4^ WT or MIF KD 4T1 cells were implanted in the mammary fat pad of female Balb/c mice. Mice were treated with CD4/8 depleting antibodies starting 2 days before tumor implantation and every 4 days thereafter. **A,** Tumor size was monitored starting at day 8 by caliper measurement. **B,** Tumors were harvested and weighed at day 20 of tumor growth. n = 6 mice per group. One-way ANOVA. * p<0.05, ** p<0.01, **** p<0.0001.

In order to confirm that T cells are required for control of MIF KD tumors, we performed a T cell depletion experiment by treating WT and MIF KD 4T1 tumor-bearing mice with CD4 and CD8 depleting antibodies or isotype control antibodies (cIgG) throughout the course of tumor growth. Given that both CD4 and CD8 T cells exhibited enhanced IFNgamma production in MIF KD tumors indicating enhanced functionality, we hypothesized that both populations are important in controlling growth in these tumors. Therefore, we depleted the CD4 and CD8 T cells simultaneously in the same mice (**Fig 3C)**. The T cell depletion was highly effective, as confirmed at the time of tumor harvest by measuring T cell numbers in the circulation **(S5 Fig).** Depletion of T cells from mice bearing MIF KD tumors (dotted triangle) restored primary tumor growth to the level of WT tumors (solid circle) throughout the course of tumor growth **(Fig 4A)**. In contrast, depletion of T cells from mice bearing WT tumors (solid triangle) had a minimal, though still statistically significant, effect on tumor growth rate measured by calipers **(Fig 4A)**. However, at the time of harvest, the tumor weight difference did not achieve statistical significance **(Fig 4B)**. Nonetheless, this small difference suggests that there is some amount of T-cell mediated tumor growth control even in the setting of MIF–expressing tumors, but that this effect is very modest compared to the impact of T cells on the growth of MIF KD tumors. Cumulatively, these data confirm that the reduced growth rate of 4T1 tumors derived from cells that are depleted of MIF is dependent on an intact T cell response.

### MIF expression in the primary tumor leads to decreased dendritic cell abundance and activation in the tumor

**Fig 5:**
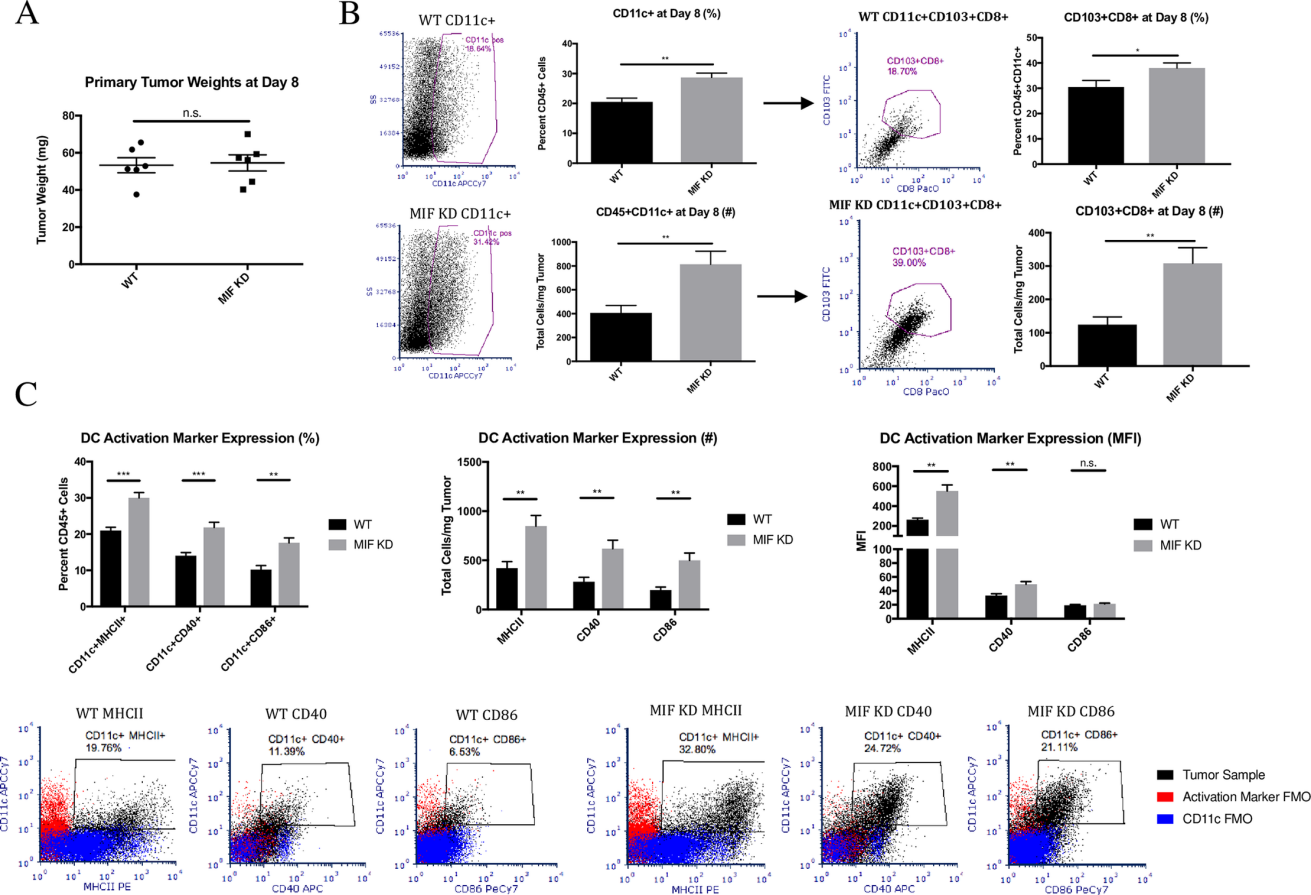
MIF expression in the primary tumor leads to decreased dendritic cell abundance and activation in the tumor. 1.0 x 10^4^ WT or MIF KD 4T1 cells were implanted in the mammary fat pad of female Balb/c mice. **A,** Tumors were harvested and weighed at day 8 of tumor growth, which is the first point at which palpable tumors are detectable. Tumors were digested and analyzed by flow cytometry for infiltration of dendritic cells by cell surface markers **(B)** and activation markers **(C)**. Representative flow plots are shown in panels B and C. n = 6 mice per group. One-way ANOVA. * p<0.05, ** p<0.01, *** p<0.001.

We next hypothesized that the increased T cell abundance and activation in MIF KD tumors is due to enhanced activation of dendritic cells (DCs) either in the draining lymph nodes or the tumor. To test this, we examined WT and MIF KD 4T1 tumor-bearing mice for the presence and activation status of tumor-infiltrating and lymph node DCs. Because the difference in activated T cells was apparent at day 10, we examined DCs at day 8, as peak DC activation would be expected to occur slightly before T cell activation. No difference in primary tumor size was observed between WT and MIF KD tumors at this time point **(Fig 5A)**. We also did not observe a statistically significant increase in total tumor-infiltrating leukocytes based on CD45 staining at this time point **(S6 Fig)**. However, the abundance of CD11c+ DCs was increased in MIF KD tumors. Within this CD11c+ population, we also observed an increase in an unexpected population of CD103+CD8+ DCs in the MIF KD tumors **(Fig 5B)**. We also examined the activation state of intratumoral DCs based on expression of MHCII, CD40 and CD86, with all gating determined based on the FMO controls shown in flow plots in **Fig 5C**. MIF KD tumors contained both a greater percentage of activated DCs, as well as a greater number per mg of tumor when compared to MIF expressing tumors **(Fig 5C)**. DCs in MIF KD tumors also had higher expression of these activation markers on a per-cell basis as quantified by mean fluorescence intensity (MFI) **(Fig 5C)**. The gating strategy for the described DC analysis is shown in S7 Fig **(S7 Fig)**. In contrast to the observation in tumors, the draining lymph nodes of mice bearing WT or MIF KD tumors showed no differences in DC numbers or activation status **(S8 Fig)** Taken together, these data suggest that MIF depletion results in an increased abundance and activation of DCs specifically in the tumor.

### MIF-expressing tumor cells show decreased markers of immunogenic cell death under serum-free conditions

**Fig 6:**
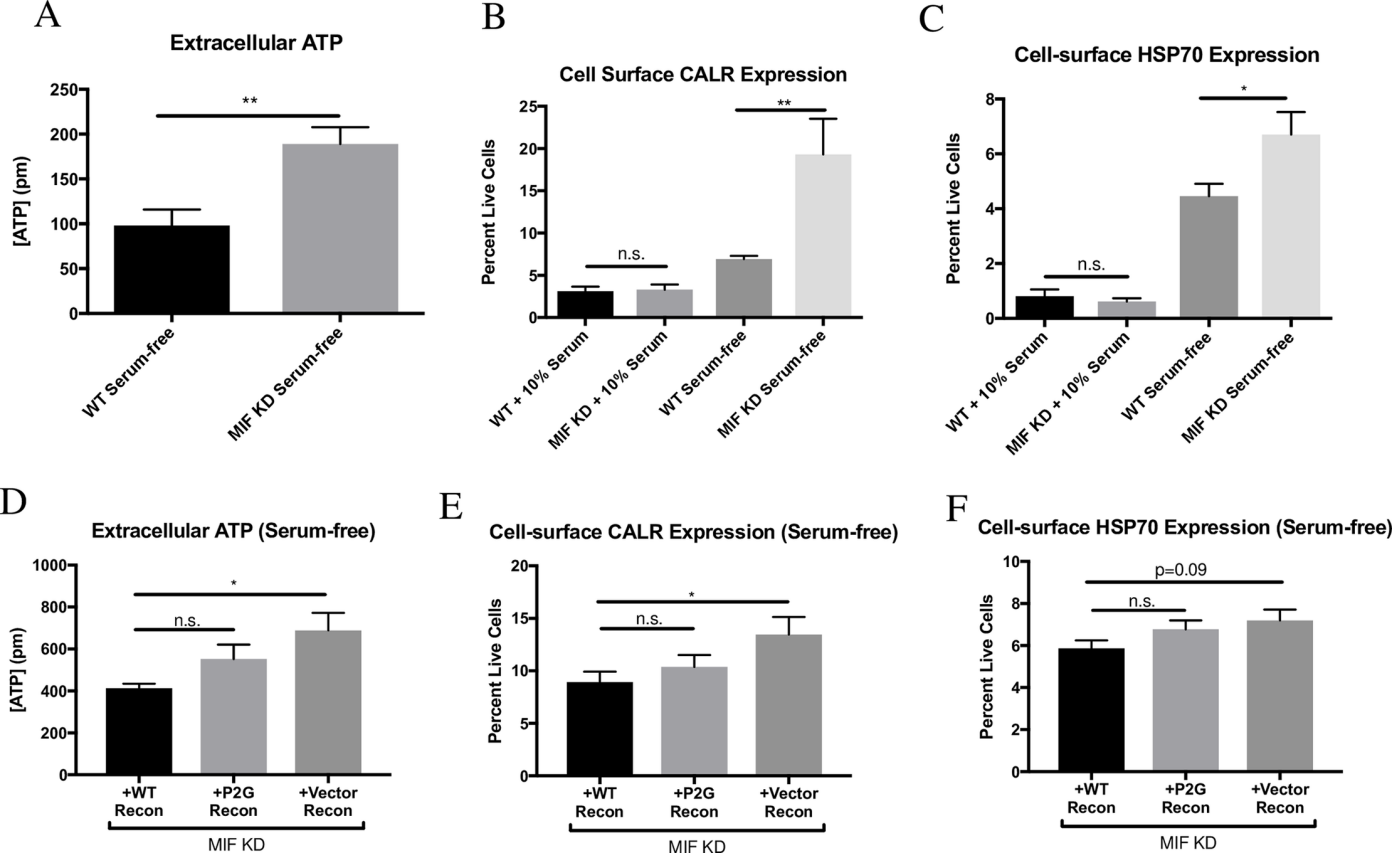
MIF-expressing tumor cells show decreased markers of immunogenic cell death under serum-free conditions. **A-C,** WT or MIF KD 4T1 cells were grown in 10% serum-containing media overnight, and then media was replaced with fresh 10% serum or serum-free media. After 24 hours, media was sampled and tested for extracellular ATP. After 48 hours, cells were harvested and stained by flow cytometry for cell surface expression of calreticulin or HSP70. **D-F,** MIF KD 4T1 cells reconstituted (recon) with WT MIF, or P2G MIF, or with an empty vector as a control were grown in 10% serum-containing media overnight, and then switched to serum-free media. After 48 hours, media was sampled and tested for extracellular ATP and cells were harvested and stained by flow cytometry for cell surface expression of calreticulin or HSP70. Data shown are the means of 3 independent experiments, with 3 replicates per experiment. **A,** Student’s t-test, **B-F**, one-way ANOVA. * p<0.05, ** p<0.01.

Recent literature has characterized a specialized type of cell death, termed “immunogenic cell death” (ICD), in which the cell death process itself renders cancer cells susceptible to detection by the immune system [26,35,36]. We hypothesized that loss of MIF expression in the 4T1 cancer cells would promote ICD, which could explain the enhanced immune response observed in the MIF KD tumors. Several standard markers of ICD have been established, including extracellular ATP release and exposure of calreticulin (CALR) and certain heat-shock proteins, including HSP70, on the cell surface [25–27]. When cultured under serum-free conditions, we found that MIF KD cells exhibit an increase in all of these markers of ICD when compared to MIF expressing cells **(Fig 6A–6C)**. This suggests that depletion of MIF promotes ICD when cells are exposed to challenging growth conditions. Reconstitution of MIF KD cells with WT MIF reduced the expression of these ICD markers in serum-free culture conditions **(Fig 6D–6F)**, demonstrating that the phenotype is due to loss of MIF expression. Interestingly, reconstitution of MIF KD cells with the mutant P2G MIF resulted in an intermediate phenotype, suggesting that the enzymatic activity of MIF is only partially responsible for protection from the ICD response observed under serum-free conditions **(Fig 6D–6F)**.

## Discussion

**Fig 7:**
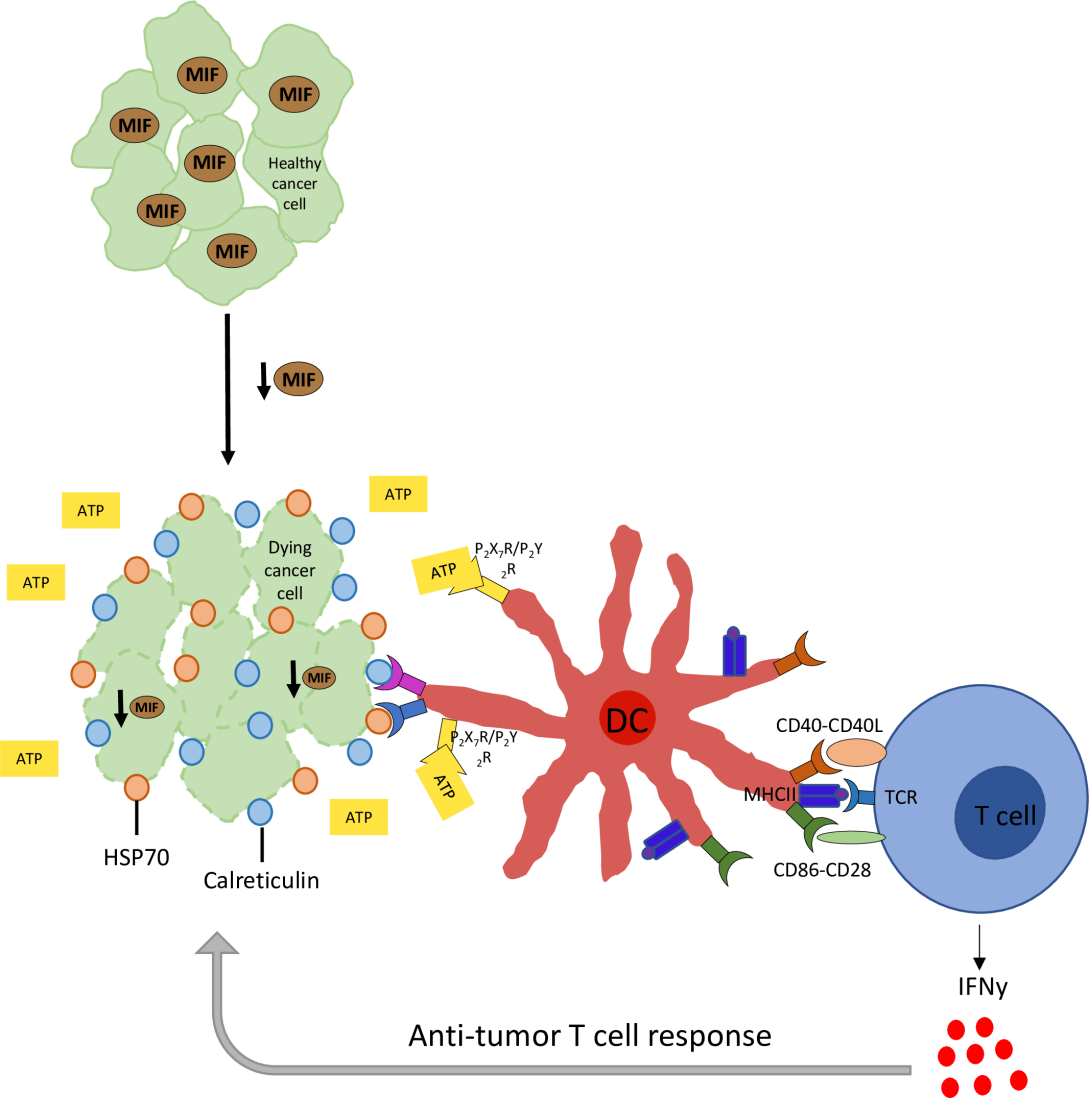
Experimental model. Upon loss of MIF expression in the tumor, cancer cells undergo ICD. This leads to an increased abundance and activation of DCs in the tumor microenvironment, resulting in an enhanced T cell-mediated anti-tumor immune response through secretion of IFNgamma in the tumor microenvironment.

Our results demonstrate that tumor cell-derived MIF is responsible for several aspects of the anti-tumor immune response in tumor-bearing animals. Specifically, MIF expression in the tumor cells reduces the abundance of activated DCs in the tumor microenvironment, and also suppresses the increase in IFN-gamma producing CD4+ and CD8+ T cells within the tumor. *In vitro*, MIF expression prevents ICD and also suppresses markers of apoptosis in serum-starved tumor cells. Based on these observations, we propose a model **(Fig 7)** whereby MIF-deficient 4T1 cells undergo ICD, leading to an enhanced abundance and activation of DCs in the tumor microenvironment. This results in an enhanced T cell-mediated anti-tumor immune response and better control of tumor growth, leading to the observed reduction in overall tumor burden observed in mice bearing MIF-deficient tumors. Using the MMTV-PyMT model, we observed that MIF-deficiency lead to a reduction in overall tumor burden as well as a delay in tumor occurrence, further supporting the role of MIF in promoting tumor growth. The corollary of this model is that an evolving tumor can overcome the growth suppressing anti-tumor effects through mechanisms that increase MIF expression. This is in agreement with the common observation that expression of MIF correlates with tumor aggressiveness in patients.

In the setting of pathogenic infection, ICD has likely evolved as a mechanism to alert the immune system to infection. Death of a small number of infected cells can lead to a robust immune response based on expression of damage-associated molecular patterns (DAMPS), recognized by cells such as dendritic cells, monocytes, and macrophages [24]. ER chaperones such as calreticulin and certain heat-shock proteins, including HSP70, are exposed on the cancer cell surface, serving as DAMPs or “eat me” signals to be recognized by the immune system [25–27]. Extracellular ATP released by cells undergoing ICD interacts with the immune system by signaling through purinurgic receptors on antigen-presenting cells (APCs) [24]. Activation of these receptors on APCs initiates the signaling processes involved in IL-1beta secretion, which is further responsible for inducing IFNgamma-producing CD8+ T cells and the anti-tumor immune response [37].

As introduced above, ICD is induced by a number of mechanisms, including treatment with certain classes of chemotherapeutics, radiation therapy, and photodynamic therapy (PDT) [23]. Our work suggests that when combined with MIF-depletion, removal of serum from *in vitro* culture conditions can also induce ICD. We hypothesize that removal of serum *in vitro* more closely mimics the stressful tumor microenvironment experienced by cancer cells *in vivo*. Therefore, our observations suggest that ICD could be induced by inhibiting MIF, which would represent another means to increase tumor immunogenicity beyond the previously identified therapeutic approaches.

MIF overexpression has been observed in a number of human cancer types, and several reports support MIF’s role in protection from apoptosis *in vitro*, including in models of lung cancer and cervical adenocarcinoma [38,39]. Recently, Johler *et al*. demonstrated that MIF expression is induced *in vitro* in Rhabdomyosarcoma (RMS) cell lines upon treatment with cytotoxic agents such as Doxorubicin, Vincristine and Etopiside, further linking MIF to the cell stress response [40]. Therefore, MIF overexpression may be a protective mechanism used by cancer cells to prevent cell death and overcome the immune response under the stressful conditions experienced within the tumor microenvironment.

Several previously published studies also strongly link MIF expression to dampened anti-tumor immune responses in cancer, which may be indirect evidence of the suppression of ICD. In a neuroblastoma model, MIF inhibits T cell activation *in vivo* [22,41], and MIF inhibition has been shown to promote conversion of melanoma patient-derived MDSCs into a more DC-like phenotype *in vitro* [21]. Similarly, addition of recombinant MIF to immature DC cultures differentiated from CD14+ monocytes has been shown to inhibit both DC maturation and migration *in vitro* [20]. In addition, we have previously published that MIF expression in the primary tumor leads to an increased abundance of intratumoral monocytic myeloid-derived suppressor cells (MDSCs), contributing to establishment of an immunosuppressive microenvironment [17]. However, this study focused on tumor-infiltrating MDSCs in late-stage 4T1 tumors, and we do not see a MIF-dependent difference in MDSCs at the early time points used in the current study. This suggests that MIF may be involved in two distinct mechanisms leading to immunosuppression in the tumor microenvironment depending on the time of tumor growth. All of these studies, when taken together, suggest that MIF is an important mediator of the immunosuppressive tumor microenvironment.

We have demonstrated that, in the 4T1 model, loss of MIF expression leads to a robust increase in activated DCs intratumorally by day 8 of tumor growth. Within the CD11c+ DC population, we observed an increase in an unusual population of dendritic cells expressing both CD103 and CD8 in mice bearing MIF KD tumors. A similar population of DCs has been previously characterized in the spleen as being highly efficient at phagocytosis of circulating apoptotic cells, as well as cross-presentation of antigens [42]. While this population of cells was shown by Qiu et al. to be tolerogenic in the setting of the spleen, this population of cells is not well-described intratumorally, and may serve as a mechanism of T cell activation via antigen uptake and presentation in the tumor microenvironment.

At day 10 of tumor growth, the MIF-deficient tumors contain significantly more IFNgamma-producing CD8+ and CD4+ T cells. IFNgamma has been established as a critical cytokine involved in the trafficking of activated T cells from the draining lymph nodes to the tumor [43], differentiation of cytotoxic immune cell subsets capable of direct tumoricidal activity [44], as well as directly inducing tumor cell growth arrest [45–47]. This suggests that the observed increase in IFNgamma-producing T cells in the tumor microenvironment may explain the decreased growth of the MIF-deficient tumors in immune competent animals. We confirmed that the T cells are important by demonstrating that simultaneous depletion of CD4 and CD8 T cells restored the growth of MIF KD tumors to parallel that observed in MIF-expressing tumors. Further dissection of the relative contribution of CD4 versus CD8 cells to the MIF-dependent immune-mediated control of tumor growth will be of interest in future studies.

Our work proposes a novel mechanism through which MIF controls cancer growth and progression through manipulation of the host immune system. When combined with earlier work by our group and others, this suggests inhibition of MIF may be a valuable therapeutic approach. Combination of a potent MIF inhibitor with any of the promising immunotherapy options already in the clinic, or those in the developmental pipeline could lead to robust, long-lasting immunity in the setting of cancer.

## Supporting information

S1 Fig Confirmation of MIF depletion and re-expression in 4T1 cell lines. MIF expression was detected by immunoblot in cell lysates from **A,** WT and MIF KD 4T1 cell lines and **B,** in the MIF KD cell line reconstitued with either WT MIF, P2G MIF or a vector control.
(PDF)

S2 Fig Confirmation of MIF status in WT and MIF KO MMTV-PyMT mice. **A,** MIF gene deletion (top) and the MMTV-PyMT transgene (bottom) were detected by PCR. A MIF heterozygous mouse is shown as a control (top). **B,** MIF expression in WT and MIF KO mice was confirmed by immunoblot of lysate prepared from whole lung tissue.
(PDF)

S3 Fig MIF expression protects against cleaved caspase 3-mediated cell death *in vitro* in serum-free conditions. WT or MIF KD 4T1 cells were grown in 10% serum-containing media overnight, and then switched to fresh 10% serum-containing media or serum-free media for a further 48 hours. Lysates were prepared and immunoblots were performed to quantify cleaved caspase 3 expression. Data are from one experiment with 3 replicate samples and the data shown is representative of 3 independent experiments. Student’s t-test. ** p<0.01.
(PDF)

S4 Fig Gating strategy for T cell analysis by flow cytometry. Single cells were selected first, followed by gating out of cellular debris by FSC vs. SSC. Next, dead cells were excluded by live/dead viability dye. T cells were gated using CD3 positivity. CD4+ and CD8+ subsets were gated, and IFNgamma positivity was assessed within each T cell subset. All populations gated on FMOs as shown.
(PDF)

S5 Fig Treatment of mice with CD4/8 depleting antibodies leads to almost complete loss of CD4/8+ T cells in the circulation during tumor growth. 1.0 x 10^4^ WT or MIF KD 4T1 cells were implanted in the mammary fat pad of female Balb/c mice. Mice were treated with CD4/8 depleting antibodies starting 2 days before tumor implantation and every 4 days thereafter. Blood was harvested on day 20, at the time of tumor harvest and analyzed by flow cytometry for the presence of **A,** CD4+ and **B,** CD8+ T cells. Cells were pre-gated through live, CD45+ and CD3+ parameters. n = 6 mice per group. One-way ANOVA. * p<0.05, **** p<0.0001.
(PDF)

S6 Fig MIF expression in the primary tumor does not affect total intratumoral leukocyte abundance. 1.0 x 10^4^ WT or MIF KD 4T1 cells were implanted in the mammary fat pad of female Balb/c mice. Tumors were digested and analyzed by flow cytometry for infiltration of leukocytes using the cell surface marker CD45. Student’s t-test revealed no statistically significant differences in CD45+ cell abundance as a function of MIF expression in the tumor.
(PDF)

S7 Fig Gating strategy for DC analysis by flow cytometry. Single cells were selected first, followed by gating out of cellular debris by FSC vs. SSC. Next, CD45+ cells were selected, followed by CD11c+ positive cells. CD8+ and CD103+ DC subsets were gated through CD11c+ cells. MHCII, CD86 and CD40 activation markers were gated through CD11c+ cells as well. All populations gated on FMOs as shown.
(PDF)

S8 Fig MIF-depletion in the primary tumor does not affect dendritic cell presence or activation in the draining lymph node. 1.0 x 10^4^ WT or MIF KD 4T1 cells were implanted in the mammary fat pad of female Balb/c mice. Draining (inguinal) and non-draining (axillary) lymph nodes were harvested at day 8 of tumor growth. Lymph nodes were dissociated and analyzed by flow cytometry for infiltration of dendritic cells by **A,** cell surface markers and **B,** activation markers. A non-tumor bearing naïve mouse was used as a control. n = 6 mice per group. One-way ANOVA.
(PDF)
