# Supplementary figures and images for: Macrophage Migration Inhibitory Factor protects cancer cells from immunogenic cell death and impairs anti-tumor immune responses
Source: PLoS One. 2018 Jun 4;13(6):e0197702. doi: 10.1371/journal.pone.0197702 (PMC5986154; doi:10.1371/journal.pone.0197702)

A

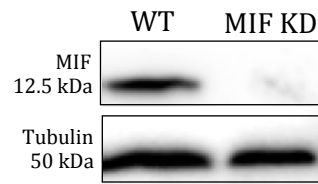

B

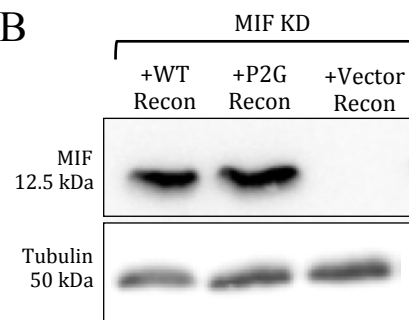

Supplement: S1 Fig — MIF expression was detected by immunoblot in cell lysates from A, WT and MIF KD 4T1 cell lines and B, in the MIF KD cell line reconstitued with either WT MIF, P2G MIF or a vector control. (PDF) [file pone.0197702.s001.pdf]

A

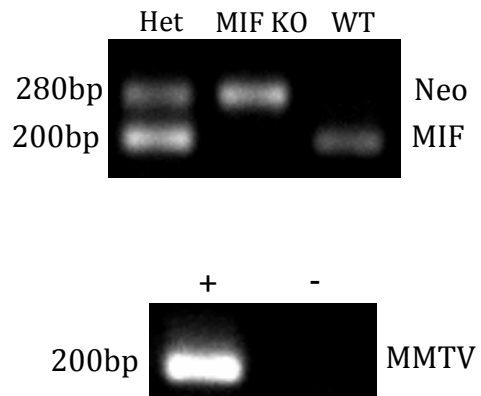

B

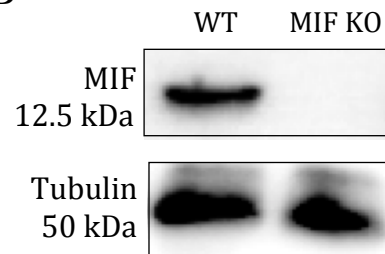

Supplement: S2 Fig — A, MIF gene deletion (top) and the MMTV-PyMT transgene (bottom) were detected by PCR. A MIF heterozygous mouse is shown as a control (top). B, MIF expression in WT and MIF KO mice was confirmed by immunoblot of lysate prepared from whole lung tissue. (PDF) [file pone.0197702.s002.pdf]

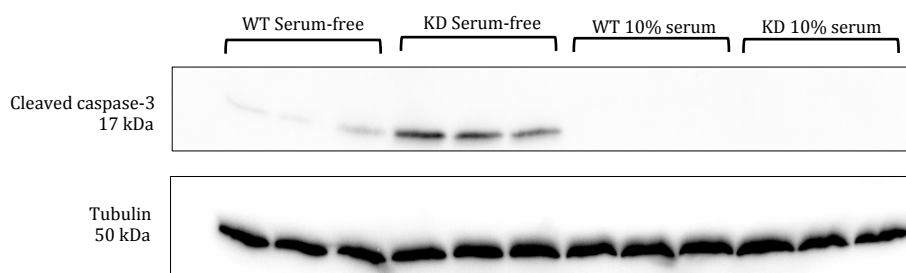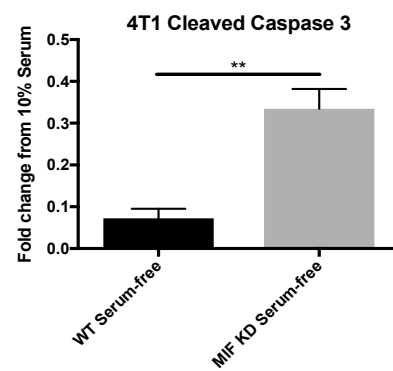

Supplement: S3 Fig — WT or MIF KD 4T1 cells were grown in 10% serum-containing media overnight, and then switched to fresh 10% serum-containing media or serum-free media for a further 48 hours. Lysates were prepared and immunoblots were performed to quantify cleaved caspase 3 expression. Data are from one experiment with 3 replicate samples and the data shown is representative of 3 independent experiments. Student’s t-test. ** p<0.01. (PDF) [file pone.0197702.s003.pdf]

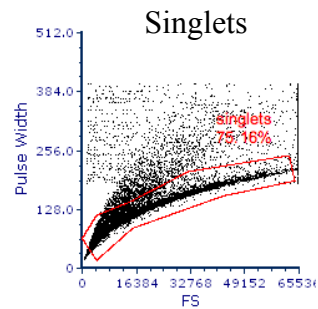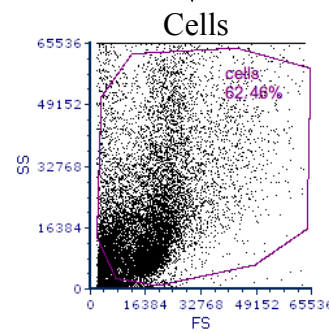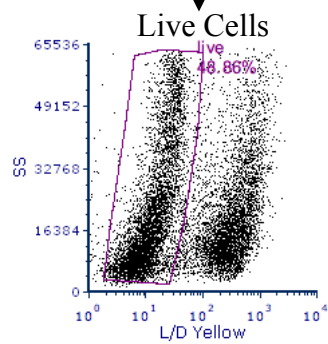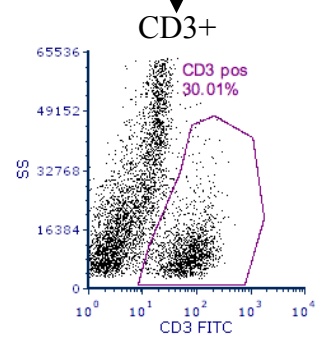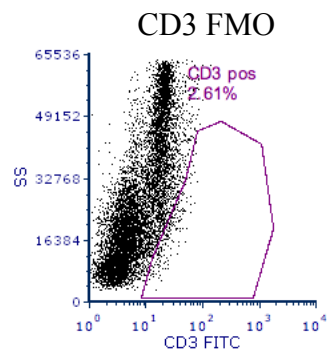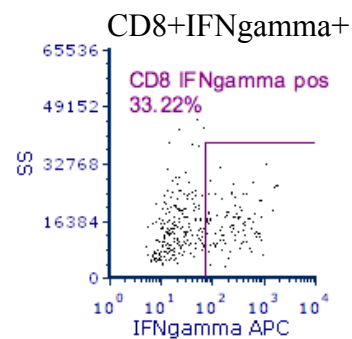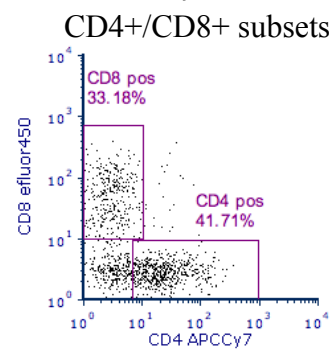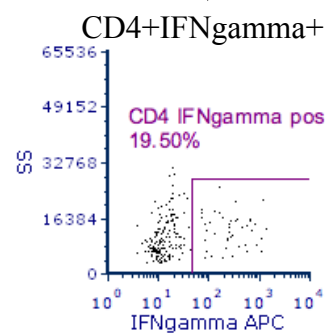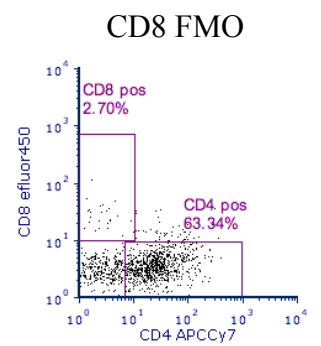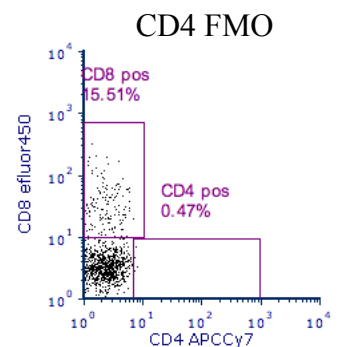

Supplement: S4 Fig — Single cells were selected first, followed by gating out of cellular debris by FSC vs. SSC. Next, dead cells were excluded by live/dead viability dye. T cells were gated using CD3 positivity. CD4+ and CD8+ subsets were gated, and IFNgamma positivity was assessed within each T cell subset. All populations gated on FMOs as shown. (PDF) [file pone.0197702.s004.pdf]

A

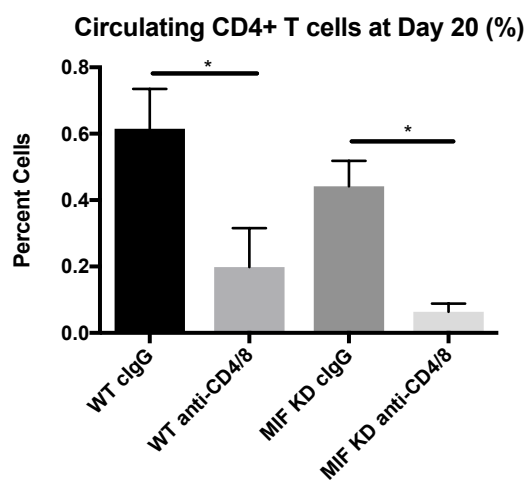

B

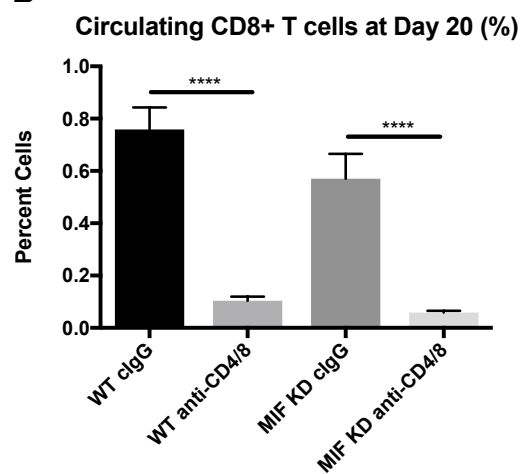

Supplement: S5 Fig — 1.0 x 10^4^ WT or MIF KD 4T1 cells were implanted in the mammary fat pad of female Balb/c mice. Mice were treated with CD4/8 depleting antibodies starting 2 days before tumor implantation and every 4 days thereafter. Blood was harvested on day 20, at the time of tumor harvest and analyzed by flow cytometry for the presence of A, CD4+ and B, CD8+ T cells. Cells were pre-gated through live, CD45+ and CD3+ parameters. n = 6 mice per group. One-way ANOVA. * p<0.05, **** p<0.0001. (PDF) [file pone.0197702.s005.pdf]

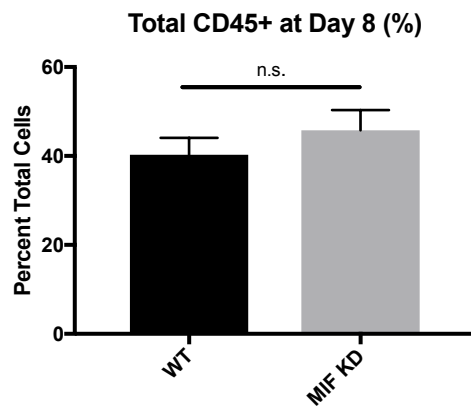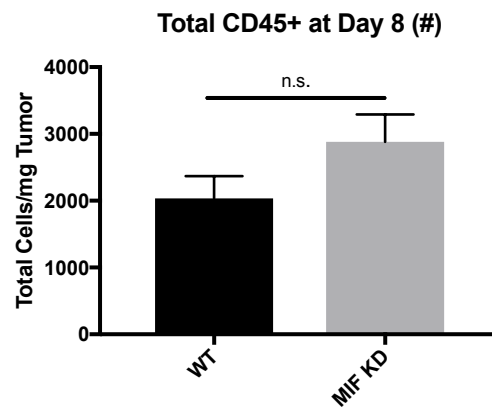

Supplement: S6 Fig — 1.0 x 10^4^ WT or MIF KD 4T1 cells were implanted in the mammary fat pad of female Balb/c mice. Tumors were digested and analyzed by flow cytometry for infiltration of leukocytes using the cell surface marker CD45. Student’s t-test revealed no statistically significant differences in CD45+ cell abundance as a function of MIF expression in the tumor. (PDF) [file pone.0197702.s006.pdf]

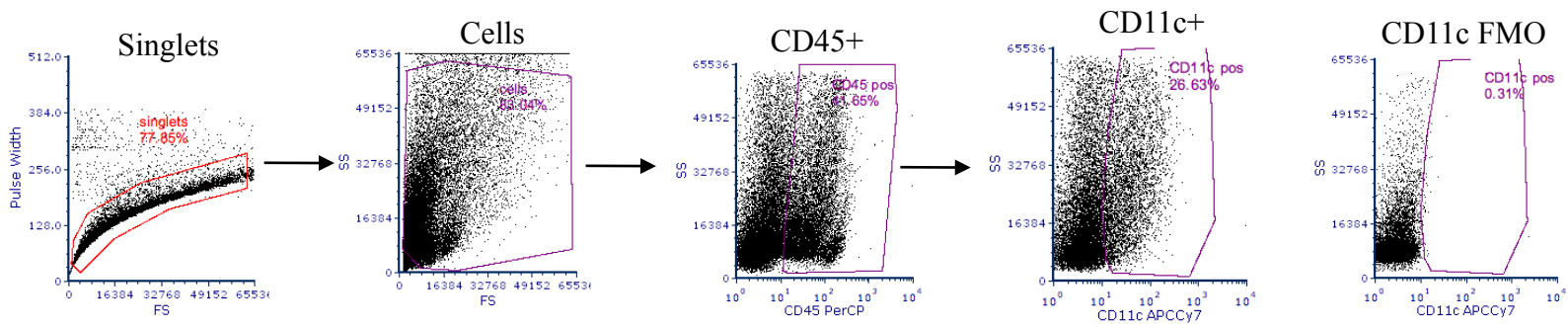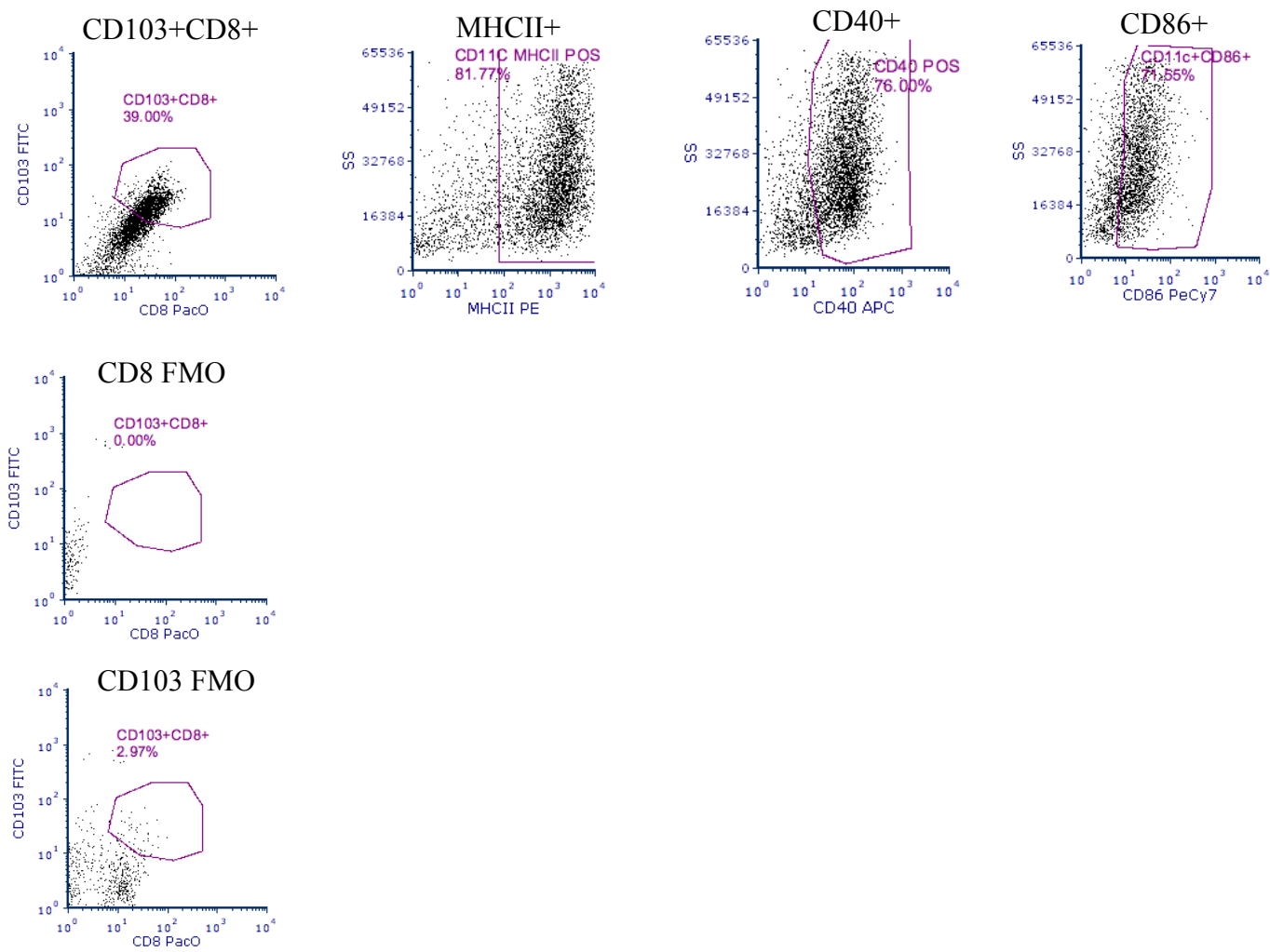

Supplement: S7 Fig — Single cells were selected first, followed by gating out of cellular debris by FSC vs. SSC. Next, CD45+ cells were selected, followed by CD11c+ positive cells. CD8+ and CD103+ DC subsets were gated through CD11c+ cells. MHCII, CD86 and CD40 activation markers were gated through CD11c+ cells as well. All populations gated on FMOs as shown. (PDF) [file pone.0197702.s007.pdf]

A

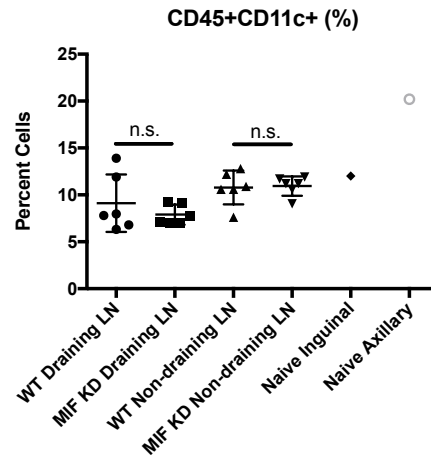

B

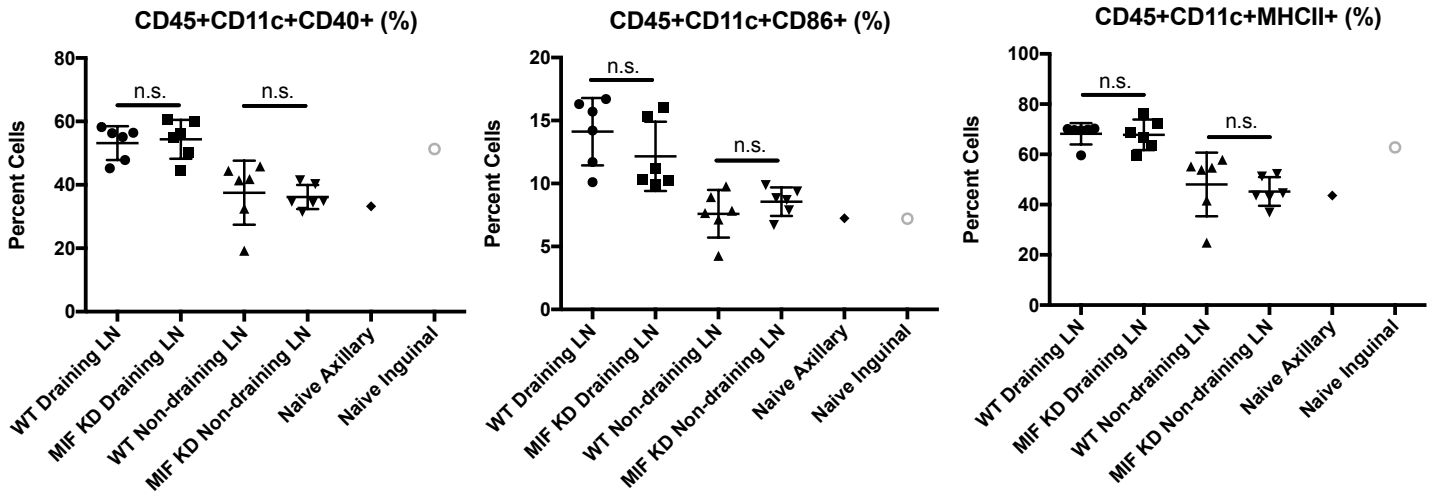

Supplement: S8 Fig — 1.0 x 10^4^ WT or MIF KD 4T1 cells were implanted in the mammary fat pad of female Balb/c mice. Draining (inguinal) and non-draining (axillary) lymph nodes were harvested at day 8 of tumor growth. Lymph nodes were dissociated and analyzed by flow cytometry for infiltration of dendritic cells by A, cell surface markers and B, activation markers. A non-tumor bearing naïve mouse was used as a control. n = 6 mice per group. One-way ANOVA. (PDF) [file pone.0197702.s008.pdf]
